# Investigating the comorbidity of COPD and tuberculosis, a computational study

**DOI:** 10.3389/fsysb.2023.940097

**Published:** 2023-02-23

**Authors:** Cheryl L. Sershen, Taha Salim, Elebeoba E. May

**Affiliations:** ^1^ Department of Biomedical Engineering, University of Houston, Houston, TX, United States; ^2^ Department of Medical Microbiology and Immunology, University of Wisconsin-Madison, Madison, WI, United States

**Keywords:** chronic obstructive pulmonary disease (COPD), *Mycobacterium tuberculosis*, comorbidity, macrophage immune response, agent-based model (ABM), granuloma, multi-scale modeling, lung diseases

## Abstract

Recent research has shown that people who suffer from chronic obstructive pulmonary disease (COPD) have a greater propensity to contract and develop tuberculosis (TB) than the general population. Not only is the hazard ratio for contracting active tuberculosis triple that of the general population for those with COPD, but that the probability of death from any cause during the first year was double that of the tuberculosis population as a whole. This observation suggests that patients with COPD are less likely to progress to latent tuberculosis infection (LTBI) and are more likely to develop active tuberculosis than the general population. While similar susceptibility rates to TB are known to occur in populations with other ailments of the lung, particularly HIV, emphysema or asthma, patients with COPD (both emphysema and chronic bronchitis) are statistically more at risk for the disease. To examine the comorbidity effects of COPD on tuberculosis disease and granuloma formation, the process by which *Mycobacterium tuberculosis* (Mtb) is either contained or disseminates, we used a multi-scale model that integrates pathophysiological and immunopathological aspects of COPD and TB. Depicting chronic obstructive pulmonary disease smoker and non-smoker populations, we integrate agent-based models (ABM) of cellular immune response, physiological models of pulmonary capacity for COPD smoker/non-smoker, systems biology models of macrophage immune response to Mtb, and metabolic models to capture intracellular and extracellular Mtb metabolism and proliferation. We use our model to investigate key drivers of disease outcomes of clearance, granuloma-based containment, and disseminated disease in individuals with COPD and TB for smoking and non-smoking populations.

## Introduction

Chronic obstructive pulmonary disease (COPD) is associated with lower respiratory tract infections and considered the fourth leading cause of death in the United States (Xu et al., 2020). COPD is a combination of emphysema and chronic bronchitis, weakening the immune system through constant systemic inflammation. Individuals with COPD are susceptible to multiple co-morbidities, such as heart failure, diabetes, atherosclerosis, osteoporosis, muscle wasting, and also co-infections. For those with COPD, active tuberculosis (TB) becomes a high risk consequence of exposure to *Mycobacterium tuberculosis* (Mtb), with recent studies showing that compared to individuals without COPD, those who suffer from COPD have triple the risk of developing active TB after COPD has been established. Once contracted, the COPD/TB individual is twice as likely to die from disease or other causes (Inghammar et al., 2010).

COPD and TB have common risk factors, including smoking and socioeconomic status, and both result in significant extracellular matrix and consequential tissue damage in the lungs. However, causal links between these related pathologies remain difficult to determine. Using computational modeling, we investigate mechanistic drivers in the immunological and immune-modulated physiological response of the COPD/TB patient that lead to active disease. Globally millions of people are exposed or infected with Mtb, resulting in nearly a quarter of the population having latent TB infection (LTBI) (Ding et al., 2022), understanding the differential drivers that increases active disease versus the outcome of LTBI in COPD/TB individuals could provide insight on treating COPD/TB comorbidity.

Several agent-based models (ABM) of tuberculosis infection have been developed and used to capture the spatio-temporal dynamics of granuloma formation, as well as systemic impact of infection (Segovia-Juarez et al., 2004; Ray et al., 2009; Datta et al., 2016; Marino et al., 2018; Wessler et al., 2020; Joslyn et al., 2022); see (Kirschner et al., 2017) for an extensive review of TB-related models. Models have accounted for cellular interactions and several have notably incorporated and investigated intracellular models of key reactions in macrophage immune response (Marino et al., 2015), Mtb metabolism (Pienaar et al., 2016; Sershen et al., 2016), impact of oxygen in the physiological environment (Datta et al., 2016; Sershen et al., 2016) and cellular interactions (Evans et al., 2020; Hult et al., 2021; Millar et al., 2021). These models leverage the extensibility of the ABM platform to investigate aspects of TB granuloma formation and its role in disease.

In prior work, we developed a multiscale, agent-based model (ABM) of oxygen mediated granuloma formation during TB infection and disease (Sershen et al., 2016), which can be further extended to investigate the impact of comorbid conditions on TB disease. Using our model, we correlated the physiological immune response to infection, changes in the microenvironment of the lung parenchyma, and Mtb metabolic adaptation to the outcome dynamics of Mtb clearance, active tuberculosis, and granuloma-mediated LTBI (Sershen et al., 2016). By incorporating physiological drivers and pathogen-specific response for the average, healthy population upon exposure to Mtb, we were able to replicate the Mtb infection outcome distribution reported for *in vivo* primate studies (Gideon et al., 2015). In our current work we expand our physiologically-based ABM (ABM-PHYS) model of Mtb infection to capture the pathophysiological damage and immune dis-regulation observed in COPD individuals and use the model to replicate the predominant outcomes of COPD/TB comorbidity. We compare the distribution of COPD/TB disease outcomes to the distribution of outcomes seen in non-COPD individuals who tend towards an LTBI outcome and investigate differences in response between non-smokers and smokers with varying severity of COPD related damage. As a secondary focus we investigate intracellular response of infected macrophages within the non-smoker COPD context. Due to the damage associated with smokers, the total macrophage pool susceptible to infection (number of resting macrophages) is less for the smoker population than for the non-smoker populations. Additionally, given the non-triviality of expanding the ABM-PHYS model in this manner and the added computational cost, we elected to focus our study on infected macrophages in non-smokers and demonstrate feasibility.

## Materials and methods

Our ABM-PHYS model was used to explore the full range of parameters representing TB infection in the average “healthy” non-COPD individual, and the range of parameters produced approximately the same distribution of outcomes comparable to a primate model of TB infection, disease and LTBI (Sershen et al., 2016). Using data from clinical and empirical studies of COPD, we expanded the parameter space for our original ABM-PHYS and incorporated new model components to capture COPD specific parameters and components, including: over recruitment of macrophages and T cells, pulmonary dynamics such as emphysema in COPD individuals, cytokine and chemokine variations, and the effects of metalloproteinases on disease (Butler, 1976; Frank et al., 1997; Saetta et al., 1999; Sethi, 2000; Cataldo et al., 2001; Russell et al., 2002; Groneberg and Chung, 2004; O’Donnell et al., 2004; Barczyk et al., 2006; Hodge et al., 2007; Black et al., 2008; Sandland et al., 2008; Di Stefano et al., 2009; Sethi, 2010; Frankenberger et al., 2011). For each simulated individual, the Global Initiative for Chronic Obstructive Lung Disease (GOLD) stage between 1 and 4 is selected, which determines the level of tissue damage and parameter ranges for age, arterial blood gas and partial pressure of oxygen of the simulated individual (Mapel et al., 2015; Portillo et al., 2015). GOLD stage 1–4 is associated corresponds to mild, moderate, severe, and very severe COPD. Whether a COPD individual is a smoker is also randomly determined. Smoker/non-smoker status impacts MMP9 secretion levels and tissue damage. Table 1 contains new and updated parameter ranges used in the COPD model; ranges for non-COPD individuals and parameters not directly associated with COPD in the model may be found in (Ray et al., 2009; Sershen et al., 2016) [Table 1 and Appendix 4/Table 1 in (Sershen et al., 2016)]. We varied these parameters across the specified ranges and used uncertainty quantification and sensitivity analysis methods to evaluate the phenotypically-grouped outcomes, which were classified using our pre-defined heuristic [Appendix 1 in (Sershen et al., 2016)].

**TABLE 1:** COPD associated parameters for the ABM-PHYS. Ranges correspond to upper and lower bounds used in the Latin hypercube sampling (LHS) based sensitivity analysis to identify model drivers.

Parameter description	COPD range	Units	Source, notes
GOLD stage (severity)	(1–4)		Mapel et al. (2015) (T3); using the distribution found by mMRC
age	(49–82.5)	Years	Portillo et al. (2015) (T1); using distributions by GOLD stage
arterial blood gas (oxygen)	(42.75–77)	mmHg	Portillo et al. (2015) (T1); using distributions by GOLD stage
alveolar-arterial oxygen partial pressure difference	(12.5–34)	mmHg	Portillo et al. (2015) (T1); using the distributions by GOLD stage
prob.recruit.mac	(0.06, 0.60)		Frankenberger et al. (2011); macrophage recruitment increases 6-fold
prob.recruit.T	(0.01, 0.20)		Saetta et al. (1999); recruitment of CD8^+^ cells doubles
			Hodge et al. (2007)
			Tzanakis et al. (2003)
pulmonary.blood.source	(2.45e + 08, 9.43e + 08)	Molecules	Dock et al. (1961)
residual.volume	(3.38e + 08, 1.328e + 09)	Molecules	Corriveau et al. (1989)
smoker	(0, 100)		CDC (2015); smoker if *p* < 39
MMP9.secretion	(5, 40, 60, 100)[ns, ls, ms, hs]	ng/mL	Russell et al. (2002)
MMP9.saturation.threshold	(2500–2700)	ng/mL	Vernooy et al. (2004)
MMP9.diffusion.constant	(5.8e-09, 6.2e-9)	*cm* ^2^/sec	Collier et al. (2011)
MMP9.half-life	7 h	Not varied	Akool et al. (2003)
initial.matrix.cells.destroyed.by.COPD	(0, 10000)	Grid cell	Sershen et al. (2016)
mr reduced ability for phagocytosis of bacteria	(0–1)	Bacteria	Hiemstra (2013)
ma reduced ability for phagocytosis of bacteria	(0–5)	Bacteria	Hiemstra (2013)

The remaining parameters related to the functioning of ABM-PHYS were previously reported in (Sershen et al., 2014; Sershen et al., 2016). Abbreviations: Global Initiative for Chronic Obstructive Lung Disease (GOLD), non-smoker (ns), light smoker (ls), medium smoker (ms), heavy smoker (hs), resting macrophage (mr), activated macrophage (ma).

### Dynamics of breathing with COPD

Although total lung capacity in individuals with COPD tends to be greater than that of the general population, the lung tissue thickens due to extracellular matrix remodeling, resulting in increased occurrence of fibroids in the lung (Butler, 1976; Ohno et al., 2008; Sandland et al., 2008). Forced expiratory volume in 1 s falls to about 35% of normal. Thus, while the residual volume of the lung may be larger, the COPD lung is very poorly oxygenated. Also, given the resulting tissue damage due to emphysema, there is less oxygen diffusing into the lung tissue. Additionally, the ventilation-perfusion gap (alveolar-arterial gradient) widens for those with COPD. Using the information described in Table 1, it is possible to generate an alveolar-arterial gradient and level of hypoxemia for each simulated patient. The increased ventilation-perfusion gap in the COPD individual makes oxygen transfer through damaged alveolar tissue to capillaries extremely difficult. We implemented the O2 diffusion model using steps previously described [Appendix 2 of (Sershen et al., 2016)].

To determine the amount of oxygen that pervades the alveolar tissue of the of COPD patient, we varied the alveolar oxygen tension based upon simulated parameters from Table 1, in order to determine the amount of oxygen available at a given grid point in the tissue sample. This resulted in generally lower alveolar partial pressure of oxygen, which means fewer oxygen molecules available in the alveolar tissue and thus earlier onset of hypoxic conditions upon granuloma formation than in the non-COPD patient. This is aggravated by the fact that the number of macrophages recruited is six times higher than normal with twice the T cell recruitment (thus greater oxygen consumption in alveolar tissue). These dynamics resulted in larger hypoxic regions than those arising from granuloma formation in the non-COPD patient.

#### Cytokines and chemokines in COPD

The pro-inflammatory cytokine TNF-*α* is released by T cells, neutrophils and macrophages. In COPD, there is increased recruitment of small macrophages and T cells that secrete TNF*α*, so that in the lung parenchyma a sepsis-like condition results. Since there are higher percentages of both CD8^+^ T cells and macrophages operative in COPD (Saetta et al., 1999; Hodge et al., 2007), the lung is chronically activated as a result of highly inflammatory cytokines produced by these immune cells and the increased production of enzymes that degrade the extracellular matrix. To capture the heightened inflammatory state, we increased the number of resting macrophages and increased the number of CD8^+^ cells, which are significant producers of IFN-*γ* and TNF-*α* (Saetta et al., 1999; Frankenberger et al., 2011). We also increased the percentage of T cells secreting INF-*γ* and TNF-*α*. In the model IFN-*γ* produced by T cells can act on macrophages based on the condition of the macrophage. IFN-*γ* activates macrophages proximal to *γδ*-T cells to clear their bacterial load and enable them to become efficient at phagocytosis of Mtb. T cell produced cytokines can induce apoptosis in a chronically infected macrophage where, upon cell death, there is some probability that the macrophage’s bacterial load spreads out onto the damaged lung parenchyma. The chemokines CCL2, CCL7, CCL22, and CCL13 are reported to be significantly higher for an individual with COPD (Di Stefano et al., 2009). Since our original ABM-PHYS model only inluded CCL2, we increased the chemokine production level for CCL2 by 16 fold to capture this feature of COPD.

#### Tissue remodeling *via* matrix metalloproteinases

Matrix metalloproteinases, extracellular matrix degrading enzymes, MMP9, MMP2 and MMP12 are released by macrophages and neutrophils and contribute to lung tissue remodeling and damage in COPD (Cataldo et al., 2001; Russell et al., 2002; Chakrabarti et al., 2007). By definition all COPD patients have some level of emphysema and active chronic bronchitis. Therefore in the progression to COPD a significant amount of tissue remodeling has already taken place within the lung. Alveoli have been severely damaged and possibly even destroyed. The walls of the lung have become thick and the lung parenchyma will contain areas of caseation and necrosis, which can permit bacterial growth. TNF-*α* and IFN-*γ* contribute to the production of tissue-damaging metalloproteinases by stimulating macrophages to produce MMP9, MMP2 and MMP12. The metalloproteinases work in concert to degrade and dissolve elastin. The cytokine activation cascade and consequential degradation is characteristic of emphysema. With the secretion of MMP9, MMP2, and MMP12 tissue viability is reduced even more and the metalloproteinases continue to erode the extracellular matrix during the first 200 days of infection. We modeled MMP9 expression as a concentration field and used thresholding to enable the progression of emphysema; see Table 1 (Russell et al., 2002).

To simulate the destroyed, emphysematous tissue present in the alveolar space and lung parenchyma of the COPD lung, we initialize a random number of grid cells to be non-viable at the outset of our *in silico* study. A non-viable grid cell represents necrotic tissue, which is a tissue cell location with no oxygen and cell particles are not permitted to move into non-viable grid locations. Bacteria can be deposited onto these grid cells if a chronically infected macrophage bursts or is killed as a result of TNF-*α* apoptosis and the intracellular bacterial load distributed to surrounding grid cells.

### Integrating macrophage molecular-scale response into the COPD-TB model

The macrophage proinflammatory response against intracellular pathogens such as *M. tuberculosis* (Mtb) activates a plethora of complex biochemical pathways that lead to the production of inflammatory cytokines and chemokines (Sasindran and Torrelles, 2011; Mattila et al., 20131950). To explore the potential impact of COPD and smoking-related damage on molecular-scale immune response, we extended and integrated a systems biology based macrophage model to capture intracellular immune response mechanisms. We build on our prior *in silico* macrophage model, which was used to investigate the dynamics of inducible nitric oxide synthase (iNOS) expression under TNF*α* and IFN-*γ* activation (Salim et al., 2016). Focusing on potential COPD-associated variations in response, we model proinflammatory response dynamics upon exposure to Mtb’s pathogenic lipoarabinomannan (LAM) and in the context of inflammatory cytokines from the microenvironment. Key microenvironment variables captured that influence or are produced by macrophages include: pro-inflammatory cytokines TNF*α* and IFN-*γ*; MCP1 (CCL2), key chemokine for the recruitment of macrophages; and RANTES (CCL5), a chemoattractant for T cells and various leukocytes (van Crevel et al., 2002).

We focused on the activation of MCP1 gene expression *via* the MAPK pathway following bacterial uptake or/and TNF*α* stimulation as observed in various studies (Wang et al., 2000; Deshmane et al., 2009). Both TNF*α* and LPS have been shown to activate the MAPK pathway leading to the activation of transcription factors NF-*κ*B and AP1 (Chan and Riches, 2001; Jones et al., 2007; Apostolou et al., 2013). Wang et al. also showed that the MCP1 promoter contains three NF-*κ*B, one AP1, and one sequence specific transcription factor (Sp1) binding sites with NF-*κ*B binding serving as the rate limiting step (Tan and Khachigian, 2009). The resulting MCP1 gene expression was modeled by the rate in Eq. 1.

$$\begin{array}{ll} & \frac{\partial MCP1\text{\_}mRNAn}{\partial t}\\ & \;=\frac{k_{179}\times KD2_{\mathrm{MCP1}}\times NFkBn{\left(t\right)}^{3}+k_{180}\times NF\kappa Bn{\left(t\right)}^{3}\times AP1\left(t\right)\times Sp1Pn\left(t\right)}{KD1_{\mathrm{MCP1}}\times KD2_{\mathrm{MCP2}}+KD2_{\mathrm{MCP1}}\times NF\kappa Bn{\left(t\right)}^{3}+NF\kappa Bn{\left(t\right)}^{3}\times AP1\left(t\right)\times Sp1Pn\left(t\right)} \end{array}$$ (1)

RANTES, shown to increase during innate immune response, recruits NK cells and eventually T-cells, which leads to increased lymphocyte-produced IFN-*γ* in the extracellular environment (Liu et al., 2005). The promoter of the RANTES gene contains three NF-*κ*B sites (Ray et al., 1997). IFN-*γ* response element 1 (IRF1) binds to the RANTES promoter, and without IRF1, RANTES gene expression is significantly reduced (Casola et al., 2001). RANTES gene expression was modeled by the rate in Eq. 2.

$$\begin{array}{ll} & \frac{\partial RANTES\text{\_}mRNAn}{\partial t}\\ & \;=\frac{k_{184}\times KD2_{\mathrm{RANTES}}\times IRF1\left(t\right)\times NFkBn\left(t\right)+k_{185}\times IRF1\left(t\right)\times AP1\left(t\right)\times NF\kappa Bn{\left(t\right)}^{3}}{KD1_{\mathrm{RANTES}}\times KD2_{\mathrm{RANTES}}\times \left(1+\frac{IRF2n{\left(t\right)}^{2}}{K_{IRF2}}\right)+KD2_{\mathrm{RANTES}}\times NFkBn\left(t\right)+NFkBn{\left(t\right)}^{3}\times AP1\left(t\right)\times IRF1\left(t\right)} \end{array}$$ (2)

Given COPD’s association with loss of connective tissue in the lungs, infection associated matrix remodeling and potential regulation by matrix proteins and inflammatory response proteins, we captured macrophage production of MMP-9 in our model (Lepidi et al., 2002; Woo et al., 2004; Yu et al., 2007). Monocyte chemoattractants were shown to upregulate MMP-9 production *via* the MAPK, ERK and JNK pathways, and MCP1 is able to activate both of these pathways in an autocrine and/or paracrine manner (Jimenez-Sainz et al., 2003; Yu et al., 2007). The MMP-9 promoter contains two AP1 and one NF-*κ*B binding sites with the proximal AP1 binding serving as the rate-limiting step (Ray et al., 20051950). MMP-9 gene expression was modeled by the rate in Eq. 3.

$$\begin{array}{ll} & \frac{\partial MMP9\text{\_}mRNAn}{\partial t}\\ & \;=\frac{k_{206a}\times KD2_{\mathrm{MMP9}}\times AP1\left(t\right)+k_{206b}\times AP1{\left(t\right)}^{2}\times NFkBn\left(t\right)}{KD1_{\mathrm{MMP9}}\times KD2_{\mathrm{MMP9}}+KD2_{\mathrm{MMP9}}\times AP1\left(t\right)+AP1{\left(t\right)}^{2}\times NF\kappa Bn\left(t\right)} \end{array}$$ (3)

The expanded intracellular model of macrophage response included LAM activation of immune response pathways, and the biosynthesis of MCP1, RANTES, and matrix MMP-9. Gene expression was modeled using Michaelis-Menten kinetics, and LAM activation and receptor complex formation and signaling were modeled using mass-action kinetics. Sensitivity analysis and optimization were performed using the freely available software DAKOTA (Sandia National Laboratories) and partial regression correlation coefficients as in our prior model (Adams et al., 2010; Salim et al., 2016).

Using the Python Matlab engine, we integrated the macrophage model into the larger multi-scale ABM-PHYS-COPD model structure. Replacing the rule-based cell-scale model, the systems biology based macrophage model receives input based on the local environment, which includes remaining chemokine and cytokine concentrations after diffusion and degradation in the cell compartment, and bacterial levels in the extracellular space (LAM). Using these as initial conditions, the intracellular model is executed and used to determine the relative amount of chemokine/cytokine proteins to secrete into its local grid cell. Secreted proteins are added to the appropriate extracellular field every 10 min of simulation time.

The macrophage intracellular model was executed for all infected macrophages. Cell states were tracked for all macrophages that entered one of five center cell locations that form a Von Neumann neighborhood around the center grid point.

## Results

Using our expanded ABM-PHYS-COPD model we simulate the outcome of Mtb infection for a total of 63 COPD hosts with reduced pulmonary capacity and varying levels of emphysematous pulmonary damage. About 46% of the virtual COPD patients experienced at least moderate to mild hypoxemia (30 mmHg–80 mmHg). This is consistent with levels of 55.36 ± 4.01 reported for hypoxemic COPD individuals in Table 1 in (Saglam et al., 2015). Our results focus on COPD and the impact of smoking versus non-smoking on granuloma formation and function. Detailed results for healthy individuals were previously discussed in (Sershen et al., 2016).

**FIGURE 1:**
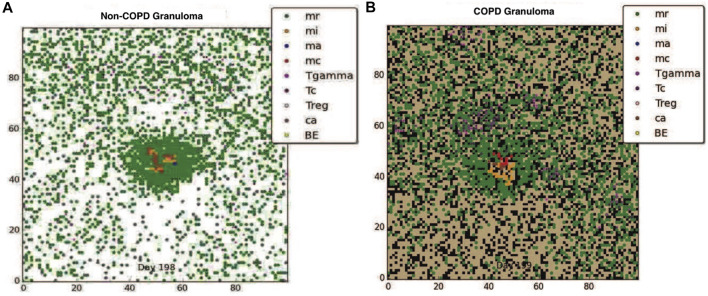
**(A)** The containment granuloma for the non-COPD individual. **(B)** The containment granuloma for an individual with COPD. Black region represents emphysematous tissue. Abbreviations: infected macrophage (mi), activated macrophage (ma), resting macrophage (mr), chronically infected macrophage (mc), T-*γ* cells (Tgamma), cytotoxic T cells (Tc), regulatory T cells (Treg), caseous tissue grid cells (ca), extracellular bacteria (BE) (Segovia-Juarez et al., 2004; Ray et al., 2009; Sershen et al., 2016).

Although clearance is the target outcome of an Mtb infected host, for hosts with comorbidity conditions such as COPD, given the reduced probability of clearance due to immune compromise, containing the pathogen within a granuloma structure is the next best alternative. Using our model we compared the impact of COPD on granuloma formation following Mtb infection. Figures 1A, B show respectively the containment granuloma for the healthy non-COPD individual and the individual with COPD/TB infection at 200 days post infection. The non-COPD host forms a granuloma consisting of a tightly packed ring of resting macrophages, which are able to effectively contain the bacteria, whereas the COPD hosts forms a granuloma that is loosely packed and containing a higher number of chronically infected and infected macrophages, as well as *γδ*-T cells. The resulting structure of the COPD granuloma is partly due to the presence of dead emphysematous tissue in the COPD host. The COPD granuloma may prove less effective in containing the Mtb infection longterm due to higher number of Mtb harboring macrophages, hence the COPD individual’s propensity towards progressing to reactivated TB disease is elevated.

### Impact of COPD and smoking on response to Mtb

We divided our 63 simulated COPD Mtb infection outcomes into four groups based on the level of tissue damage in the host, which was randomly determined for each simulation, according to GOLD stage: heavy smokers, light smokers, non-smokers with high initial damage tissue damage, and non-smokers with low initial tissue damage. Resulting groups and the number of simulated individuals per group are: non-smoker high damage (18), non-smoker low damage (16 individuals), heavy smoker (19), light smoker (10). Average results are shown for each group.

**FIGURE 2:**
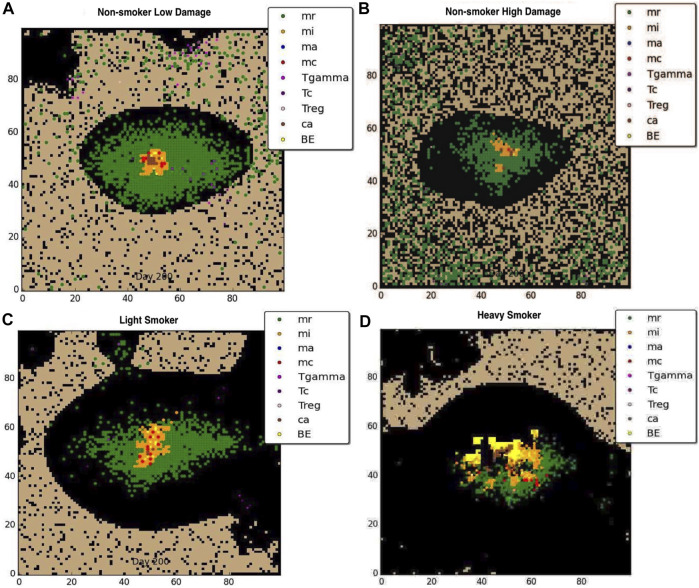
Representative granuloma for each of four categories of COPD at 200 days: **(A)** non-smoker with low damage, **(B)** non-smoker with high damage, **(C)**light smoker, **(D)** heavy smoker. Black represents emphysematous tissue. Note the extensive damage done to the ECM with continued smoking.

Figures 2A, B show the effects of previously damaged tissue (either due to prior history of smoking or other source such as breathing polluted air), for the non-smoker. Figure 2A shows a representative granuloma formed when the COPD patient does not smoke and has overall very low level of tissue damage The granuloma is clearly contained, with structure similar to the healthy LTBI infection. Figure 2B shows the granuloma formed for the non-smoker with heavy prior damage; the granuloma still contains the infection but the structure does not appear as tightly formed as in Figure 2A. Note that due to the large number of macrophages drawn to the infection site with a small but positive MMP secretion, the formation of the granuloma itself causes tissue damage, suggesting that Mtb infection may be a contributing factor to emphysema. Figures 2C, D show the effect that smoking has on the degradation of the extracellular matrix (ECM). For the light smoker the granuloma still forms but is on the verge of dissemination. In the case of the heavy smoker, the bacteria have a hospitable breeding ground and dissemination occurs. The COPD smoker’s lung parenchyma progresses to an even more emphysematous state, caused by continued secretion of MMP9 by alveolar macrophages, and ensuant MMP9 diffusion and decay caused by the metalloproteinase. The MMP9-mediated degradation illustrates a mechanism by which extracellular matrix remodeling occurs during emphysema and COPD. As the tissue becomes necrotic due to the emphysematous state, the environment in that grid cell is an area of extreme hypoxia and is inhospitable for macrophages and sustained bacterial replication. While bacteria may be spilled by an infected macrophage onto necrotic grid cells, new macrophages do not enter the categorically dead hypoxic tissue.

**FIGURE 3:**
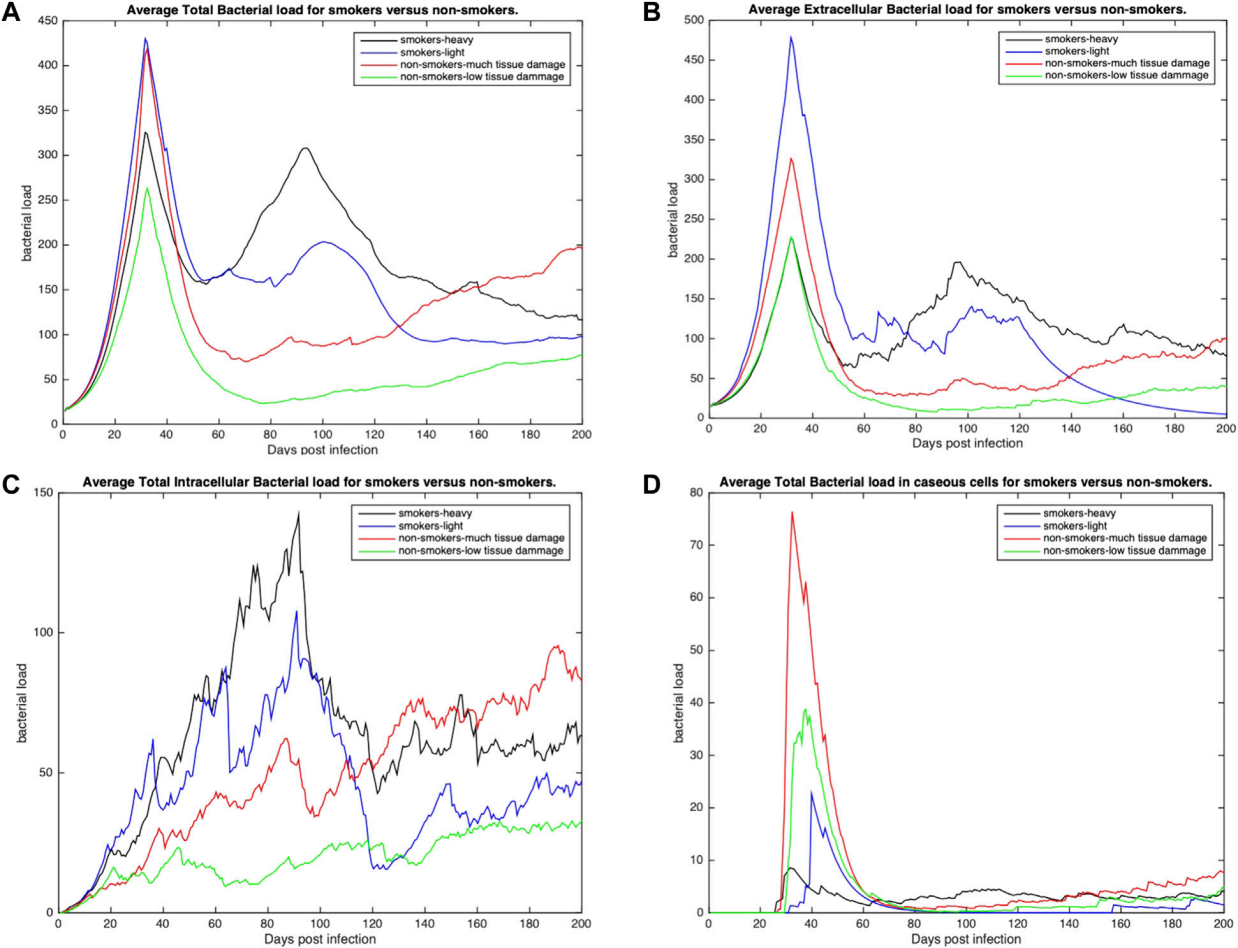
The distribution of average bacterial loads across three different environments: extracellular, intracellular and caseous cells. **(A)** Average total bacterial load for each group. **(B)** Average extracellular bacterial load across all groups (includes any bacteria in necrotic or damaged tissue). **(C)** Average intracellular bacterial load; values represent the average per infected cell across all groups. **(D)** Average bacterial load in caseous tissues. All profiles statistically significantly different using K-S test with p < 0.001.

**FIGURE 4:**
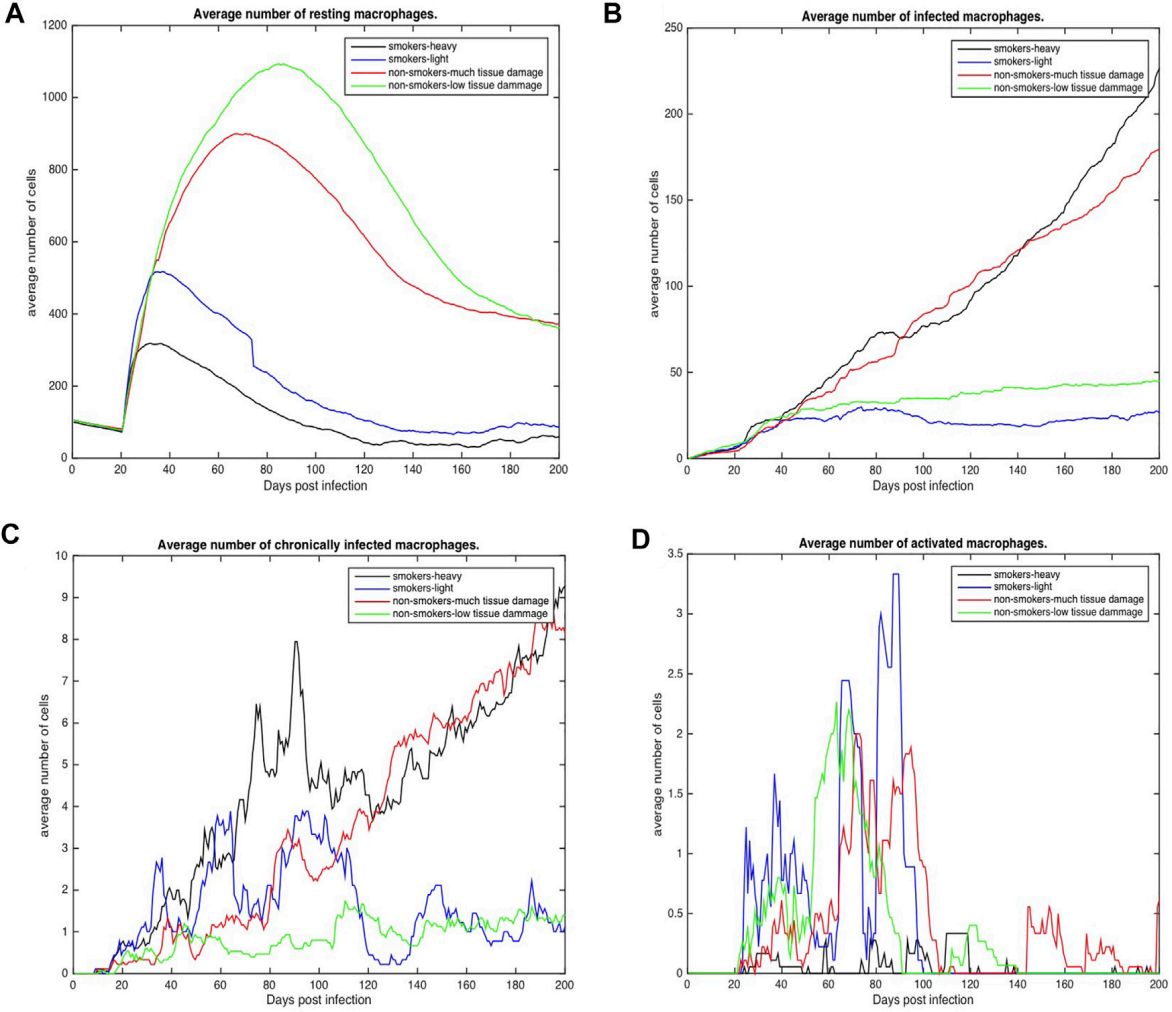
Number of macrophages involved in the immune response to Mtb for COPD non-smokers and smokers both heavy and light. Values represent the average for each COPD group. **(A)** Average number of resting macrophages across all categories **(B)** Average number of infected macrophages across all categories **(C)** Average number of chronically infected macrophages across all categories **(D)** Average number of activated macrophages across all categories.

To further understand host-pathogen mechanisms that impact the trajectory of Mtb infection in COPD smokers and non-smokers we quantitatively compared the bacterial load and cellular response of each group. Figure 3 shows the distribution of bacterial loads for four different host environments: total Mtb in the host tissue, Figure 3A; Mtb in the extracellular space, Figure 3B; Mtb in the intracellular space of macrophages, Figure 3C; and Mtb in caseous grid cells, Figure 3D. Overall, heavy smokers tend to carry higher bacterial load across all scenarios; especially as the infection continues. Non-smokers with heavy tissue damage follow. Although initially higher than non-smokers, light smokers with low tissue damage eventually reduces to below the heavy damage groups. Non-smokers with low tissue damage have the lowest bacterial loads. However the location of the bacteria differ. Initially there are significantly more extracellular bacteria in light smokers with low damage, but these are cleared nearly completely by the end of the simulation time. Non-smoking groups have more bacteria in caseous regions than their smoking counterparts, with high damage maintaining slightly more bacteria in the caseous region even near the end of the simulation. There are notable secondary peaks in total bacteria between 80 and 100 days post infection for the COPD smoker populations (Figure 3A), with a more prominent peak for smokers with heavy tissue damage. These peaks are possibly indicative of failing granulomas. Increased bacteria load corresponds to increases in chronically infected macrophages (Figure 4C) as well as increased extracellular bacteria (Figure 3B), which suggests increased macrophage cell death and potential for greater emphysematous tissue (Figures 2C, D). Results suggests that heavy smoking and high tissue damage foster higher overall bacterial loads, with damage leading to higher levels of extracellular bacteria, which can increase likelihood of disseminating disease.

**FIGURE 5:**
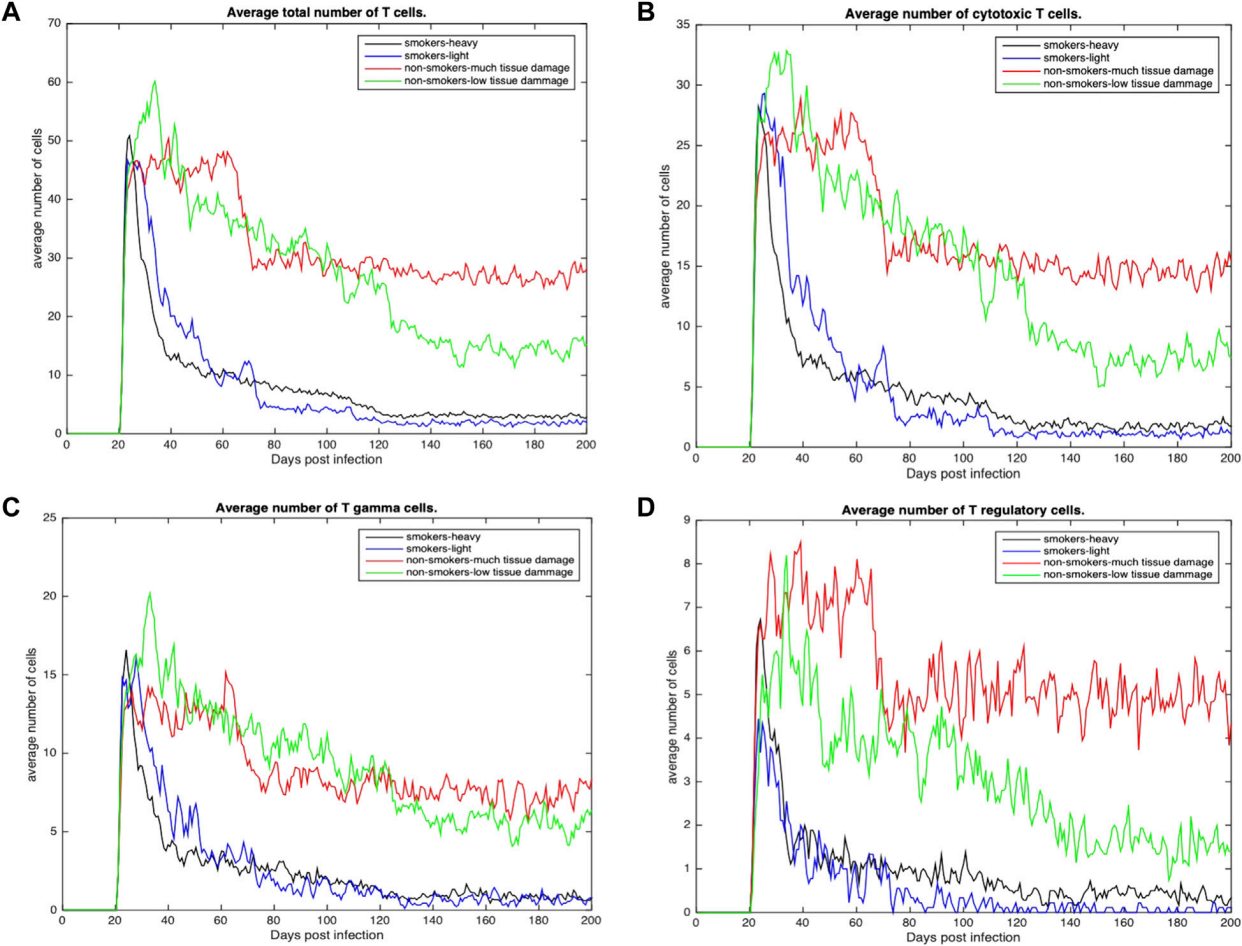
**(A)** Average total number of Tcells recruited across all categories **(B)** Average total number of cytotoxic T cells recruited across all categories. **(C)** Average total number of *γδ*-T cells across all categories **(D)** Average total number of regulatory T cells across all categories.

Figures 4, 5 show the average total numbers of macrophages and T cells for the four categorical groups. Based on the total number of macrophages (Figure 4A), smokers have a fewer number of resting macrophages indicating a harder time recruiting new macrophages. Recruitment difficulties may be due in part to the inhospitable environment of emphysematous tissue and ensuant hypoxia after granuloma formation, as well as degradation of the vasculature. Heavy smokers and those with high levels of tissue damage exhibit higher average intracellular bacteria 3(c) in infected and chronically infected macrophages Figures 4B, C. Light smokers and non-smokers with low tissue damage exhibit more macrophage activation particularly early in the simulation (prior to 100 days), when activated macrophages would be needed to help control bacteria. In Figure 4D, while there is variability over time, for the majority of the simulation non-smokers with light damage and light smoker groups have on average more activated macrophages than non-smokers with heavy tissue damage and heavy smokers. As such, macrophage function seems to deteriorate with increasing COPD-associated damage. Figures 5A–D show higher numbers of T cells recruited for non-smokers (both high and low levels of initial tissue damage). This is likely due to the fact that smoking destroys vasculature as well as tissue, hence recruiting is inhibited. Heavy damage non-smokers have higher levels of T cells across every category, with comparable numbers to low damage non-smokers only for *γδ*-T cells.

#### Characterization of infection outcomes and critical drivers of host response

Using the results of our simulation model we investigate the outcome of infection for COPD non-smoker and COPD smoker. We implement the classification heuristic outlined in (Sershen et al., 2016) to categorize simulation outcomes as either clearance, containment, or dissemination of the infection. Out of our original 63 observations (each simulation represents an observation), we eliminated simulations for COPD individuals with more than 90% non-viable lung tissue. These were removed as the significant amount of damage resulted in high level of necrotic tissue, lack of aggregate structure and difficulty effectively classifying the outcome.

Briefly, categorization relies on total/extracellular bacteria and resting macrophage levels. Clearance results when total bacteria is 0, and containment results when the change in extracellular bacteria is negative while resting macrophages are increasing. If neither a clearance or containment condition is met, then the system is classified as dissemination.

**FIGURE 6:**
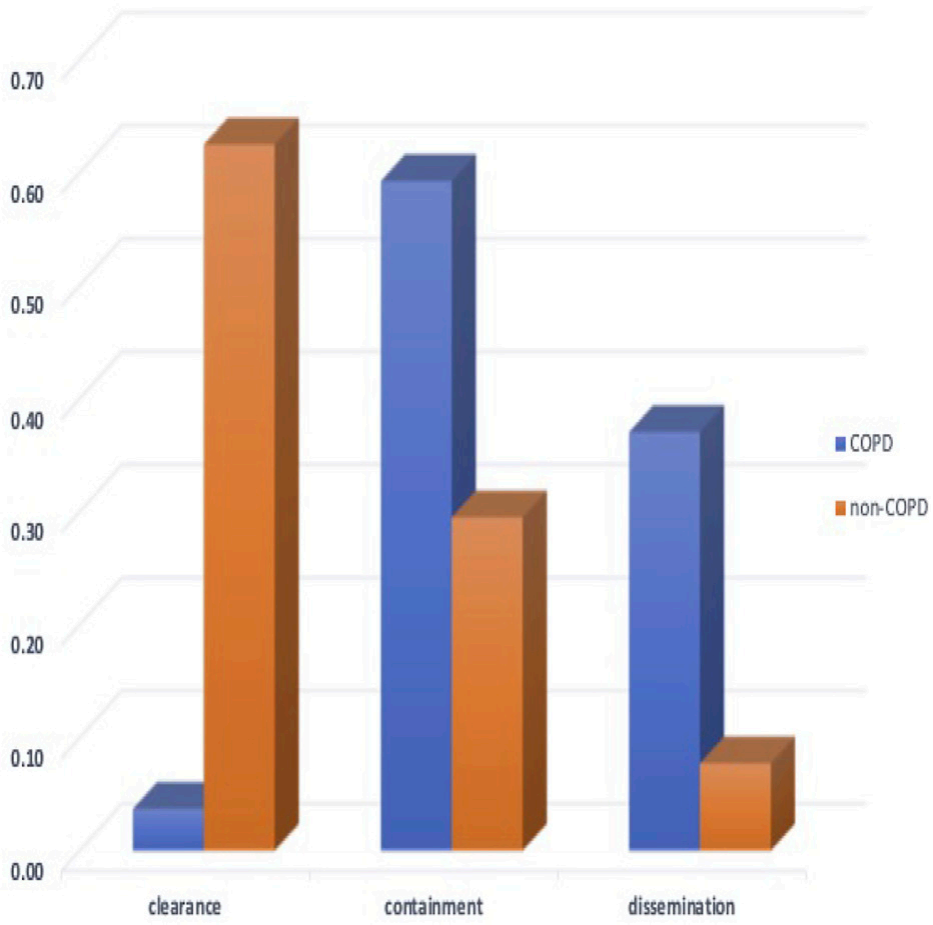
The probability distribution of qualitative outcomes for the ABM-PHYS model of Mtb infection for non-COPD individuals [which is concurrent with the distributions of outcomes for *in vivo* non-human primate models of TB using data from (Lin et al., 2014; Gideon et al., 2015)] and the distribution of qualitative outcomes for COPD individuals. The two probability distributions are significantly different (p < 0.001) with the COPD Mtb infection model resulting in approximately four times more dissemination outcomes compared to dissemination outcomes for the non-COPD scenario.

In Figure 6 we compare the distribution of simulated qualitative outcomes for the non-COPD individual infected with TB [as described in (Sershen et al., 2016)] to outcomes of infection for individuals with COPD. Two of the simulation outcomes resulted in clearance of infection. Previously the non-COPD distribution was shown to be statistically comparable to the outcome distribution observed in a non-human primate model of TB disease (Gideon et al., 2015; Sershen et al., 2016). Using a *χ* ^2^ test to compare the outcome distributions of non-COPD and COPD individuals, we find the two distributions are statistically significantly different (*p* < 0.001), with COPD individuals clearly skewing towards containment and dissemination in comparison to non-COPD model. We found that the percentage of dissemination outcomes was roughly three times (given the original simulated set) that of the healthy individual, which coincides with the finding of a triple hazard ratio of active TB disease over non-COPD individuals in (Inghammar et al., 2010).

**FIGURE 7:**
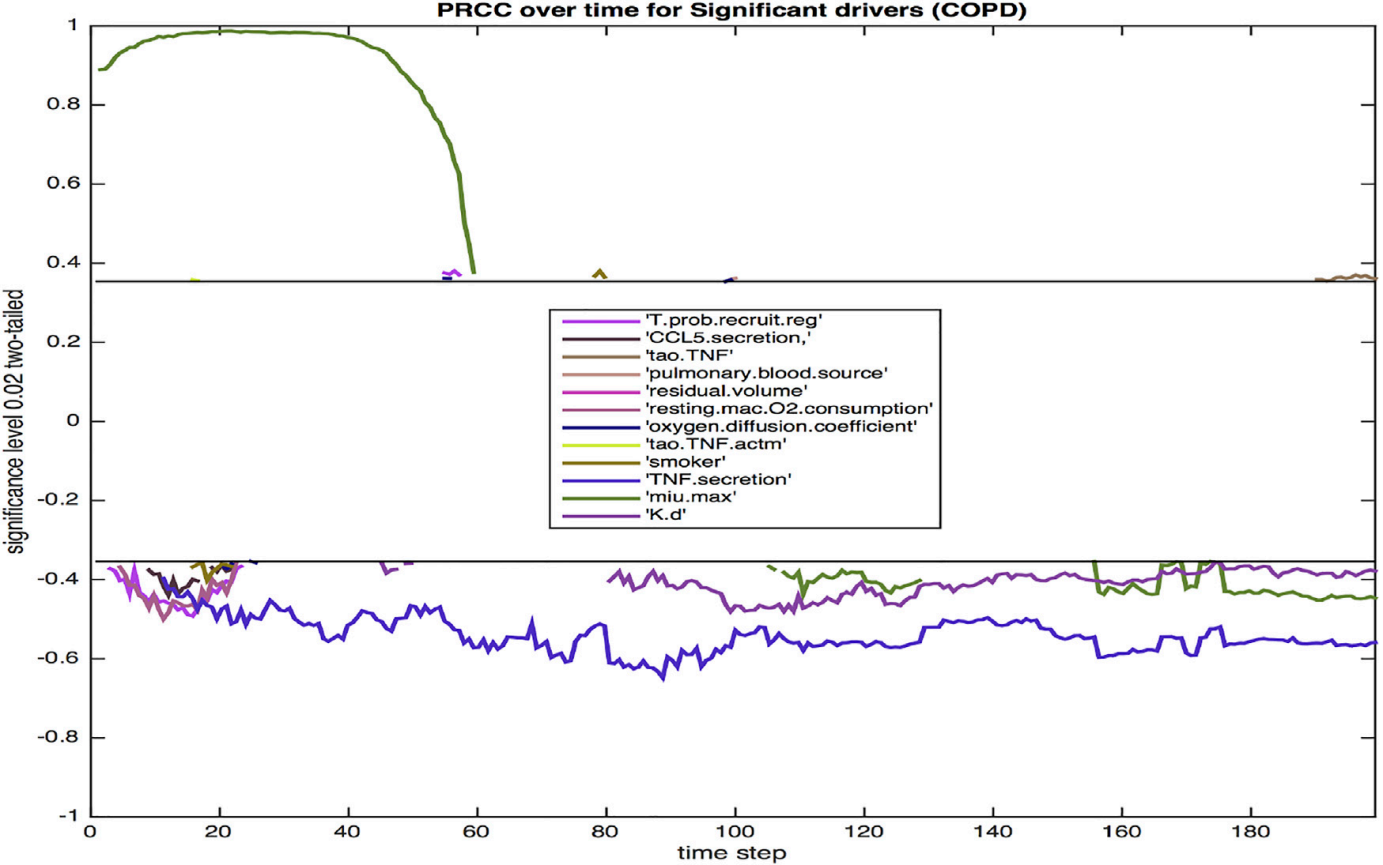
The PRCC for the model with COPD at *p* = 0.02 over the 200 day infection cycle. Parameters are defined in the Results section.

Using sensitivity analysis, we calculate the partial ranked correlation coefficients (PRCC) for our simulated COPD population to identify the most significant drivers for the observed extracellular bacterial load of the host. PRCCs are used in multi-scale models since linear coefficients do not adequately account for the non-linearities produced by stochastic modeling and logarithmic and exponential bacterial growth curves. Shown in Figure 7 are the coefficients that emerge as significant at *p* < 0.02.

Twelve parameters were identified as significant at the 0.02 level. The majority were significant during the initial 3 weeks post infection. This three-week period is a critical time frame for transition from innate to a more adaptive immune response, hence these parameters likely have a notable impact on the trajectory and outcome of immune response. Three parameters were significant throughout each phase of response (TNF*α* secretion, bacterial growth rate, bacterial death rate).

The probability of recruiting a regulatory T cell (T.prob.recruit.reg), which has a negative effect on the dependent variable of total extracellular bacteria, largely confined to the period immediately after the 10-day delay in the commencement of adaptive immunity. The amount of CCL5 (RANTES) secreted (CCL5.secretion) is negatively correlated with total bacteria, which reinforces the impact of CCL5 driven recruitment of T cells to the site of infection. Additionally, other chemokine levels are also linked to CCL5 secretion levels. The TNF threshold (tao.TNF) is positively correlated with total bacteria, since a lower threshold means more macrophages are recruited into the infection thus lowering the total bacterial load. Pulmonary blood source of oxygen (pulmonary.blood.source) and residual volume (residual.volume) are both positively correlated with total bacteria. Oxygen availability is paramount for bacterial growth, and similarly for resting macrophage oxygen consumption (resting.mac.O2.consumption). An increased level of oxygen in the macrophage increases the intracellular levels of oxygen available to intracellular bacteria. Smoking (smoker) is negatively correlated with extracellular bacteria, likely due to smoking-related increased damage of the ECM *via* increased production of MMP9. Increased ECM damage decreases the environment’s viability, making it less suited for host cells and bacterial growth.

The oxygen diffusion coefficient (oxygen.diffusion.coefficient) is mostly positively correlated with extracellular bacteria levels as fast diffusion of oxygen results in more available oxygen, which helps extracellular bacteria thrive. The threshold for TNF to activate a macrophage (tao.TNF.actm)is also positively correlated with the level of extracellular bacteria since a higher threshold implies less activation of macrophages, thus higher levels of extracellular bacteria. It is well established that TNF*α*, a key proinflammatory cytokine, is negatively correlated with total bacterial load. TNF*α* aids in macrophage activation and recruitment, stimulates production of chemokines and cytokines by macrophages, and in concert with IFN-*γ*, can activate high levels of effector molecules that lead to the death of intracellular bacteria. The negative correlation of TNF*α* secretion (TNF.secretion) with extracellular bacteria is consistent with the pro-inflammatory role of the cytokine in activating macrophages, leading to more uptake and eventual elimination of Mtb. Excessive TNF*α*, especially in the lungs of an individual with COPD can lead to septic side effects, damaging multiple organ systems. The growth rate of bacteria, *μ*-max (min.max), which determines the variable growth rate of bacteria in response to changing oxygen conditions and microenvironments, is positively correlated with total bacteria early on in the infection cycle, but becomes negatively correlated with extracellular bacteria later in the infection cycle. This transition from positive to negative correlation may be associated with increased tissue damage (Figure 3) that occurs, which limits viable tissue for host cell and extracellular Mtb occupancy, and further exacerbates COPD-associated oxygen deprivation. Reduced oxygen would particularly impact rapidly growing bacteria as demonstrated empirically and in theoretical models of Mtb growth under low oxygen environments (Wayne and Hayes, 1996; May and Sershen, 2016). Finally, a high death rate of bacteria (K.d) is associated with lower bacteria levels.

### Macrophage response for non-smokers with COPD

We integrated the macrophage molecular scale model into ABM-PHYS-COPD model to further investigate the impact of COPD on macrophage response during Mtb infection. Focusing on macrophage response in non-smokers with COPD, we simulated responses for non-smoker with high initial tissue damage and non-smoker with low initial tissue damage. We examined macrophage response for two non-smoker COPD individuals. Once infected, irrespective of location in the tissue, the macrophage response to infection was simulated, resulting in a total of 388 simulated cells, 139 macrophages from non-smoker low damage and 249 from non-smoker high damage individual. We analyze the response of cells that entered the center grid cell or locations within the Von Neumann neighborhood of the center grid during the simulation.

We evaluated the intracellular response of macrophages that enter the center grid cell at some point during the duration of the simulation. Infection is initiated in the center grid cell, with cells moving in and out of the center cell during the course of infection. Results were analyzed for cells in the Von Neuman neighborhood of the center grid as we anticipated a high likelihood of infected cells entering that grid location, resulting in observable macrophage response during the simulation.

Comparing heavy damage and low damage outcomes (Table 2), a total of two macrophages (numbered 99 and 98) entered the center grid cell (C1) in the heavy damage scenario versus four macrophages (numbered 9, 238, 110 and 439) for the low damage condition. While the initial appearance of center-grid associated macrophages were comparable (2.7 days in heavy damage versus 3.4 in low), occupancy in the center grid spanned the duration of the simulation for low damage conditions (120.5 days) but not for heavy damage conditions (27.3 days). Even when considering a grid cell in close proximity to the center cell (grid cell C2), while three macrophages (numbered 78, 15, 77) entered the proximal location their occupancy was notably less (36.3 days) than low damage conditions. With a few exceptions, under both conditions macrophage total life span was comparable and approximately 20 days. However low damage macrophages did not overlap during the simulation, but all macrophages under heavy damage conditions overlapped and co-existed during the simulation. These differences in center grid cell occupancy, time overlap and persistence of macrophages indicates that under high damage, there is a higher likelihood of tissue necrosis, preventing macrophages from entering and necrosis likely occurs earlier during the course of infection and disease. Further the level of LMN, indicating level of intracellular Mtb, increased for successive macrophages under high damage but decreased for successive macrophages under low damage conditions. This difference is indicative of bacterial dissemination and ineffective immune response in high damage conditions.

**TABLE 2:** Macrophages from non-smoking heavy and low damage COPD cells located at the center of the grid. Cells listed in order of appearance in simulation.

High damage center location (C1)				
	Cell 99	Cell 98		
LMN level at exit	0	143.6		
Sim Enter	57,600	156,000		
Sim Exit	512,400	590,250		
ΔDays	21.1	20.1		

High Damage Near Center Location (C2)				
	Cell 78	Cell 15	Cell 77	
LMN level at exit	0	73	138	
Sim Enter	90,000	368,700	432,900	
Sim Exit	541,200	518,550	784,500	
ΔDays	20.9	6.9	16.3	

Low Damage Center Location				
	Cell 9	Cell 238	Cell 110	Cell 439
LMN level at exit	38	22	1.6	0.2
Sim Enter	73,800	536,400	1,418,100	2,344,500
Sim Exit	535,800	977,700	1,976,400	2,601,900
ΔDays	21.4	20.4	25.8	11.9

**FIGURE 8:**
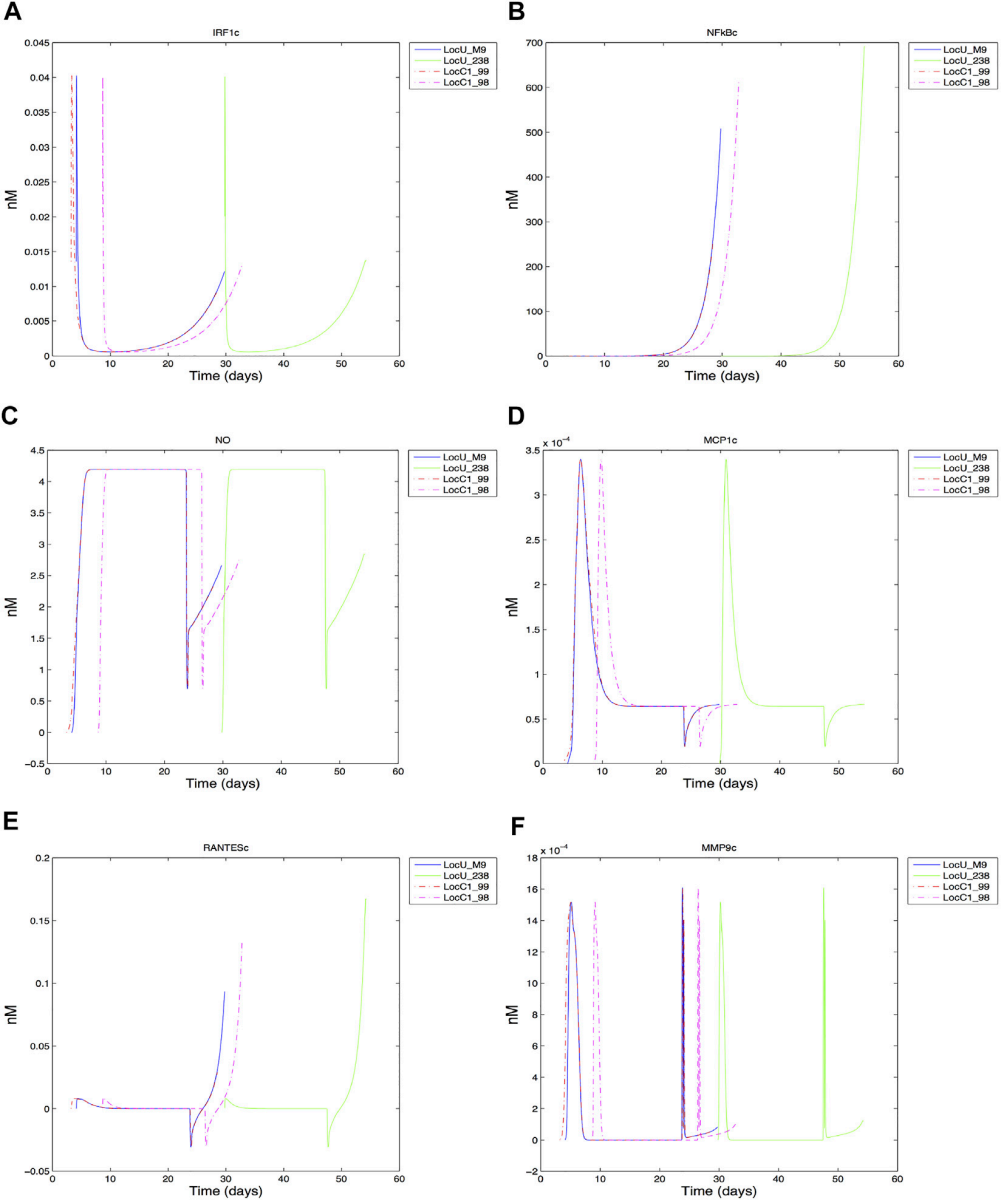
Response of center grid cell associated macrophages: **(A)** IRF1 cytoplasmic **(B)** NFkB cytoplasmic **(C)** Nitric oxide cytoplasmic **(D)** MCP1 cytoplasmic **(E)** RANTES cytoplasmic **(F)** MMP9 cytoplasmic.

Dynamics of intracellular species were compared, with the time varying cytoplasmic levels of key transcription factors (IRF1, NF-*κ*B), effector (NO), chemokiness (MCP1, RANTES), and MMP9 shown in Figure 8 (full period of time shown in Supplementary Figure S2). The general trajectory of the dynamic response of the cells are comparable for both low and high-damage non-smokers; this is likely due to the common grid location of the cells. We expect cells located in other regions exhibit different dynamics based on their respective pro-inflammatory microenvironment (Salim et al., 2016). However, we did observe some differences even when limiting our analysis to centrally located grid cells. The early expression of IRF1c contributes to the production of NO, with the later expression of NF-*κ*B helping to sustain NO levels. MCP1, macrophage chemoattractant, reaches peak production early in comparison to the T cell chemoattractant, RANTES, which peaks at the end of the macrophage lifespan. Although the general shape of intracellular species are comparable under heavy (LocC1) and low damage (LocU), the significant overlap between heavy damage macrophages makes their effective intracellular contribution similar to that of the first of four low damage macrophages responding to Mtb infection in the center cell. Much of the time period of the first macrophages in each condition overlap. There are some notable differences in peak values, with NF-*κ*B reaching a higher maximal value in macrophages in low damage conditions. During the first 30 days, cytoplasmic RANTES is highest in the second high damage macrophage to enter the center grid cell (LocC1_98) but ultimately successive low damage macrophages produce the most RANTES. There are four MMP9 peaks, which are comparable in magnitude for the four high and low damage macrophages shown. However, the time compression of peak MMP9 production within the first 30 days in high damage conditions could contribute to more rapid tissue necrosis in the center grid cell and a less effective response to infection.

## Discussion

Using our physiological-based integrated ABM model of Mtb infection, we investigated consequences of TB infection in COPD smokers and COPD non-smokers with either a moderate or high level of initial lung damage. In addition to qualitative outcomes measured with respect to bacterial load, we also considered the potential mechanistic correlations between cellular immune response, physiological parameters, and Mtb persistence. Our *in silico* experimental results clearly indicate that TB infection given the comorbidity of COPD results in an increased likelihood of dissemination (active disease) and containment (latent TB infection) as opposed to clearance, which was the more prevalent outcome for the non-COPD *in silico* models (Segovia-Juarez et al., 2004; Ray et al., 2009; Sershen et al., 2016). The COPD infection model resulted in only two cases of complete bacterial clearance out of 63 simulations with active disease (dissemination) approximately three times higher than in the non-COPD individuals, which is comparable to findings in (Inghammar et al., 2010). Continued smoking resulted in non-viable cells, increased emphysema and an abortive immune response, as evidenced by the significantly low levels of total macrophages and T cell populations. The premature cessation of the immune response enabled low levels of bacteria to remain in infected macrophages and extracellular space. We also note that increased numbers of macrophages resulted in emphysema in the locus of the granuloma, even though smoking had ceased.

A key observation from the COPD simulation study was the negative compounding effect of smoking on the immune response to Mtb for an individual with COPD. The negative correlation of smoking and tuberculosis outcome has been empirically observed (Chakrabarti et al., 2007). Smoking contributed significantly to an abortive host immune response, as it promoted increased levels of MMP9 production and accelerated the destruction of the ECM during the course of infection. The increase in non-viable tissue made the lung parenchyma inhospitable for both the immune cells and eventually the bacteria, although bacteria survived in pockets of damaged tissue and chronically infected macrophages. The excessive proinflammatory response could result in an overproduction of TNF-*α*, which while negatively correlated with bacterial load, produced sepsis-like condition and systemic host damage. Integration of the macrophage intracellular model provided new insight connecting COPD-related damage to changes in the effective timeframe and outcome of molecular-scale response in the center grid location.

Studies have found COPD as a comorbid condition, second to diabetes, in patients with tuberculosis (Chakrabarti et al., 2007). Individuals diagnosed with TB infection with COPD tend towards reduced clearance outcomes and an increase in dissemination. While it is difficult to quantify the contribution of pre-existing COPD to the development of TB disease, one study estimated COPD individuals had a 2.4 times greater risk of developing TB than non-COPD individuals (Lee et al., 2013). Smoking exacerbates host immune response in COPD patients. The granulomas for both the non-smoker highly damaged group and the light smoker are less tightly packed than the granuloma for the COPD individual with low damage. Heavy smoking more often results in a disseminated granuloma. Additionally, smoking was a significant parameter that correlated with high extracellular bacteria as was high bacterial growth rate during the initial stage of infection. These correlations highlights the importance of smoking cessation in addition to therapeutic intervention during the early stages of infection.

Overall, the model clearly demonstrates that once infected with Mtb the COPD individual is significantly more likely to have a disseminated infection or form an ineffective granuloma than the non-COPD individual. Dissemination increases the likelihood of Mtb spreading to other regions of the lung or organs outside of the lung, which is a contributory factor in the development of TB pneumonia. The inclusion of macrophage response demonstrates a viable hybrid approach for exploring the impact of COPD on dynamics of molecular immune response. However the current model has highlighted limitations with respect to model robustness in rapidly changing conditions, which can emerge during the ABM-PHYS simulations. We observed sharp changes in some of the intracellular substrate values for the macrophage model and slight negative concentrations (RANTES). These variations were not observed in the non-integrated macrophage model (Supplementary Figure S1). The differential equation-based model was optimized and validated against empirical data. However, it is highly likely that emergent dynamics of the ABM’s extracellular environment differ from initial conditions used to optimize and validate the model. In addition, the non-integrated model did not account for death or change in spatial location, both which may contribute to the edge effects observed for some substrates in the model.

Future integrated models will need to implement optimization and uncertainty quantification methods that incorporate multi-phenotype calibration approaches to increase model robustness. Extension of the model to include intracellular response of resting and activated macrophages is also needed. Continued development of integrated models that consider the physiological and immunological contributions of COPD comorbidity to TB disease can help uncover the mechanistic drivers that lead to a COPD individual’s three-times higher risk of developing active TB and two-times increased hazard ratio of dying from TB disease within the first year. Such insights can contribute to preventative interventions as well as inform therapeutic decisions in the management of COPD prior to or during TB infection.

## Data Availability

The raw data supporting the conclusion of this article will be made available by the authors, without undue reservation.

## Abbreviations

Mtb, *Mycobacterium tuberculosis*; TB, tuberculosis; LTBI, latent tuberculosis infection; TNF-*α*, tumor necrosis factor alpha; CCL2/CCL5, chemokine (C-C motif) ligand 2/5; ABM, agent-based model; LHS, Latin hypercube sampling; PRCC, partial ranked correlation coefficients; CFU, colony-forming unit; IFN-*γ*, Interferon-*γ*; COPD, chronic obstructive pulmonary disease; ECM, extracellular matrix.
