# Supplementary material for: Investigating the comorbidity of COPD and tuberculosis, a computational study
Source: Front Syst Biol. 2023 Feb 23;3:940097. doi: 10.3389/fsysb.2023.940097 (PMC12341967; doi:10.3389/fsysb.2023.940097)
Supplement: Supplementary file 1 [file DataSheet1.pdf]

**Investigating the Comorbidity of COPD and Tuberculosis, A Computational Study**

C. L. Sershen, T. Salim, E. E. May*
Department of Biomedical Engineering, University of Houston, Houston, Texas, USA
*E-mail: eemay@uh.edu

## Supplementary Materials

### *Optimization of Macrophage Intracellular Model*

Sensitivity analysis and optimization were performed using the freely available software DAKOTA (Sandia National Laboratories) in the same methodology described in Salim et al [Salim, et al. 2016; Adams, et al. 2014]. Briefly, the partial regression correlation coefficients of each specie within this model was compared against a statistically significant range obtained from Student's T-test to determine which parameter was significantly significant respective to each specie. The sum of the most significant parameters across all species was used to determine which parameters are the drivers of the system. The modified proinflammatory model results in 50 significant parameters as opposed to the 29 that were significant in our previous model.

Using the same software coupled with a genetic algorithm, we optimized the 50 significant parameters to experimental data sets of iNOS, MCP1, and RANTES [Mustafa, et al. 1998; Bjorkbacka, et al. 2004; Kopydlowski, et al. 1999]. Optimization reduced the mean squared error between iNOS mRNA and MCP1 by about 5-fold for both species (see Mean Squared Error Table). However, RANTES optimization did not give a better fit. In order to account for the different, we used a statistical regression approach where we plotted empirical RANTES relative expression as a function of *in silico* RANTES relative expression. Using an exponential regression function, we were able to obtain adjusted RANTES values by solving the equation for each value of the *in silico* output. The error for the adjusted RANTES was reduced 6-fold as opposed to the 1-fold unadjusted error values (Figure 1).

| **Mean Squared Error** | | | | |
|---|---|---|---|---|
| Species: | iNOS mRNA | MCP1 | RANTES | RANTES adjusted |
| Unoptimized | 1.0528 | 0.2126 | 1.2564 | 1.2564 |
| Set 1 | 0.2497 | 0.1022 | 1.1107 | 0.2011 |
| Set 2 | 0.2233 | 0.0963 | 1.1273 | 0.2159 |
| Set 3 | 0.1763 | 0.0622 | 1.1109 | 0.2074 |

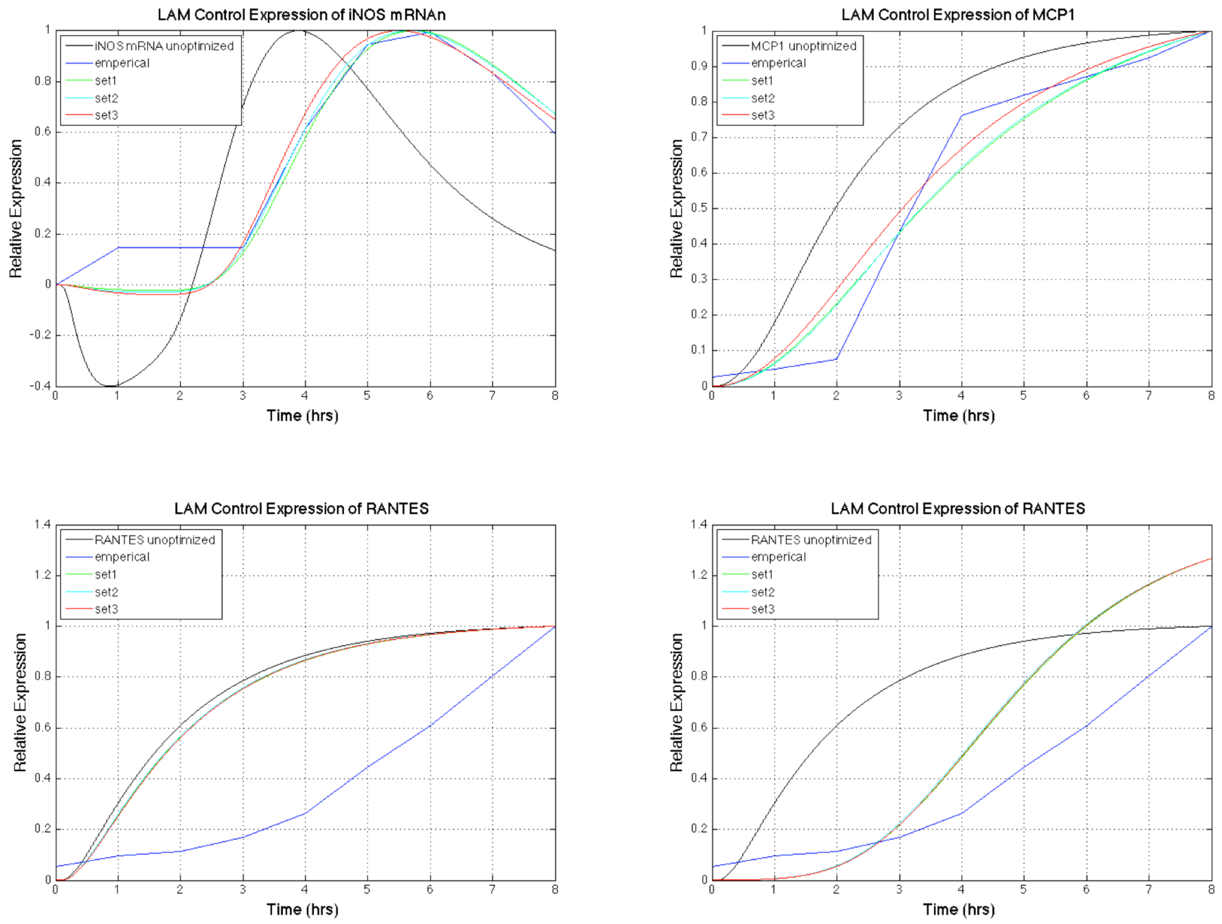


**Figure 1.** Macrophage model parameter optimization.

### *Response of Macrophages in Center Grid Cell*

We integrated the macrophage molecular scale model into ABM-PHYS model to further investigate the impact of COPD on macrophage response during Mtb infection. Below (Figure 2) are results for two macrophages (numbered 99 and 98) that entered the center grid cell (C1) in the heavy damage scenario versus four macrophages (numbered 9, 238, 110 and 439) in the low damage scenario. Results show up to 150 simulation days.

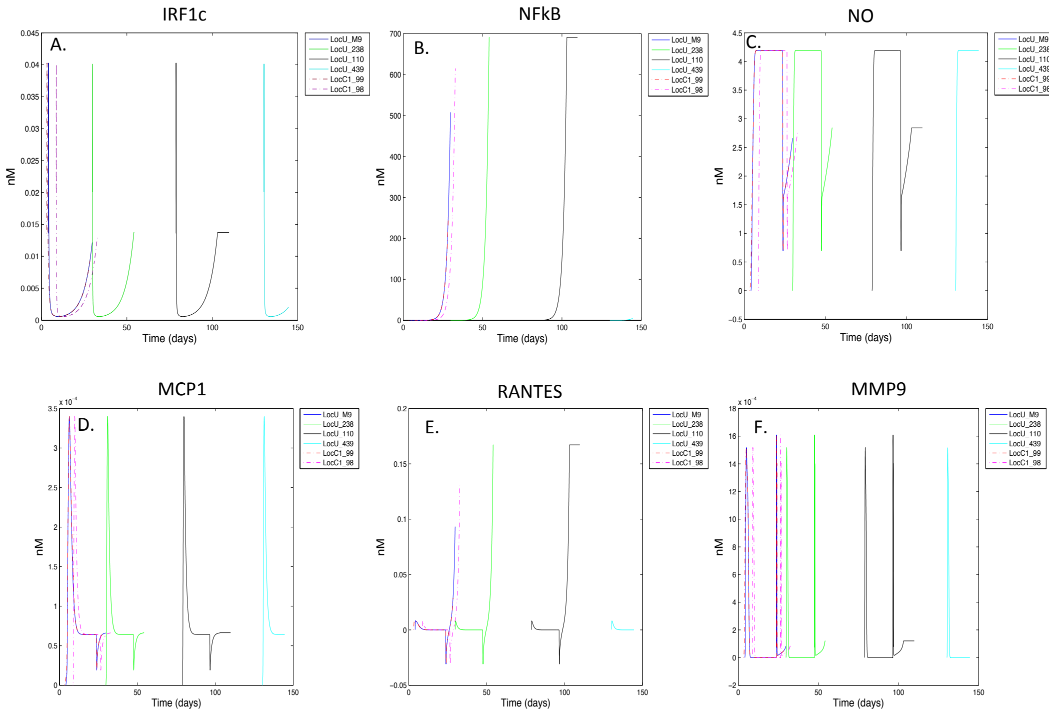


**Figure 2.** Response of center grid cell associated macrophages over 150 days simulation time: (a) IRF1 cytoplasmic (b) NFkB cytoplasmic (c) Nitric oxide cytoplasmic (d) MCP1 cytoplasmic (e) RANTES cytoplasmic (f) MMP9 cytoplasmic.

**References**

Salim T, Sershen C, May E (2016) Investigating the role of TNF-alpha and IFN-gamma activation on the dynamics of inos gene expression in lps stimulated macrophages. PloS one .

Bohnhoff, W. and Adams, B and Dalbey, K and Eddy, M JP and Gay, D. and Eldred, M (2014) Dakota, a multilevel parallel object-oriented framework for design optimization, parameter estimiation, uncertainty quantification, and sensativity analysis. Sandia Technical Report Version 5.0 user's manual, Sandia National Laboratories.

S. B. Mustafa and M. S. Olson (1998) Expression of nitric-oxide synthase in rat Kupffer cells is regulated by cAMP. The Journal of biological chemistry, 273(9).

H. Bjorkbacka and K. A. Fitzgerald and F. Huet and X. Li and J. A. Gregory and M. A. Lee and C. M. Ordija and N. E. Dowley and D. T. Golenbock and M. W. Freeman (2004) The induction of macrophage gene expression by LPS predominantly utilizes Myd88-independent signaling cascades. Physiological genomics, 19(3).

K. M. Kopydlowski and C. A. Salkowski and M. J. Cody and N. van Rooijen and J. Major and T. A. Hamilton and S. N. Vogel (1999) Regulation of macrophage chemokine expression by lipopolysaccharide in vitro and in vivo. ournal of immunology, 163(3).
